# Supplementary material for: A matching method for elderly care service personnel with multiple types of service expectations
Source: PLoS One. 2025 Feb 3;20(2):e0309419. doi: 10.1371/journal.pone.0309419 (PMC11790177; doi:10.1371/journal.pone.0309419)
Supplement: Attachment — Submitted filename: Response to Reviewers 1-2 on PONE-D-24-19546.doc [file pone.0309419.s001.doc]

**Response to the Comments of Reviewer #1 on** **PONE-D-24-19546**

We thank the referee very much for the comments and suggestions. They are very helpful for us to revise and improve the paper. The paper has been carefully revised according to the referee’s advice. We have made the following changes on the paper accordingly.

**Reviewer #1, Concern #1:** The article, especially the abstract, repeats information, regarding the goals and outcomes, which could be presented in a more condensed manner.

**Author response:** Thank you for your valuable feedback and for taking the time to review our manuscript. We sincerely appreciate your thorough evaluation, which has provided us with valuable insights to improve the quality of our work.

As suggested by the referee, the summary section has been truncated with information on repeated objectives and results.

The changes have been marked in revision mode, and the revised content is also indicated in red in the manuscript, which we have attached for your convenience.

**Reviewer #1, Concern #2:** A few terms, such as elastic service expectation type and inelastic service expectation type, could have been introduced using a definition for better understanding of the concept.

**Author response:** Thank you very much for reviewing our paper and providing valuable feedback. We sincerely appreciate your thorough evaluation, which has provided us with valuable insights to improve the quality of our work.

According to referee’s comments, we added definitions of elastic and inelastic service expectation types in the “Problem description” section to better understand this concept.

The changes have been marked in revision mode, and the revised content is also indicated in red in the manuscript, which we have attached for your convenience.

**Response to the Comments of Reviewer #2 on PONE-D-24-19546**

We thank the referee very much for the comments and suggestions. They are very helpful for us to revise and improve the paper. The paper has been carefully revised according to the referee’s advice. We have made the following changes on the paper accordingly.

**Reviewer #2, Concern #1:** The introduction is too lengthy and should be reduced, especially focused on the home-based service matching problem for elderly care.

**Author response:** Thank you for your valuable feedback and for taking the time to review our manuscript. We sincerely appreciate your thorough evaluation, which has provided us with valuable insights to improve the quality of our work.

According to referee’s comments, we have already made appropriate cuts in the “Introduction” section.

The changes have been marked in revision mode, and the revised content is also indicated in red in the manuscript, which we have attached for your convenience.

**Reviewer #2, Concern #2:** "It is evident that the matching issue faced by home care providers differs from the conventional matching issue in domestic services...." Why to assert this? What are the reasons? There are some other similar claims without reasons or references, eg., "According to a survey conducted by a few domestic help companies, elderly people typically have expectations related to their age when selecting elderly care service personnel.", "...and they typically select those with higher educational backgrounds...".

**Author response:** Thank you for reviewing our manuscript and providing valuable feedback. We sincerely appreciate your thorough evaluation, which has provided us with valuable insights to improve the quality of our work.

As suggested by the reviewer, in the description of the indicators, we added references as a basis for consideration.

*[1] Blažienė I, Žalimienė L, Between user’s expectations and provider’s quality of work: The future of elderly care in Lithuania, Journal of Population Ageing, 13 (2020), 5-23. https://doi.org/10.10 07/s12062-017-9215-1.*

*[2] Burch K A, Dugan A G, Barnes-Farrell J L, Understanding what eldercare means for employees and organizations: a review and recommendations for future research, Work, Aging and Retirement, 5 (2019), 44-72. https://doi.org/10.1093/workar/way011.*

*[3] Carpenter B D, Van Haitsma K, Ruckdeschel K, et al, The psychosocial preferences of older adults: a pilot examination of content and structure1, The Gerontologist, 40 (20000), 335-348. https://doi.org/10.1093/geront/40.3.335.*

*[4] Maiden R J, Horowitz B P, Howe J L, Workforce training and education gaps in gerontology and geriatrics: what we found in New York State, Gerontology & Geriatrics Education, 31 (2021), 328-348. https://doi.org/10.1080/02701960.2010.532749.*

The changes have been marked in revision mode, and the revised content is also indicated in red in the manuscript, which we have attached for your convenience.

**Reviewer #2, Concern #3:** Some expressions are redundant or repeated, eg., "Additionally, there may be an imbalance between the supply and demand of elderly service resources in actual life.", "It should be emphasized that there may be an imbalance between the demand and supply for senior service persons in real life,...".

**Author response:** Thank you for your valuable feedback and for taking the time to review our manuscript. We sincerely appreciate your thorough evaluation, which has provided us with valuable insights to improve the quality of our work.

As suggested by the reviewer, we have cut down on expressions that are redundant or repetitive.

The changes have been marked in revision mode, and the revised content is also indicated in red in the manuscript, which we have attached for your convenience.

**Reviewer #2, Concern #4:** The main contribution and innovation should be presented at the end of Introduction section.

**Author response:** Thank you very much for reviewing our paper and providing valuable feedback. We sincerely appreciate your thorough evaluation, which has provided us with valuable insights to improve the quality of our work.

In response to the reviewers' feedback, we add the main points of innovation at the end of the “Introduction” section.

The changes have been marked in revision mode, and the revised content is also indicated in red in the manuscript, which we have attached for your convenience.

**Reviewer #2, Concern #5:** Section 2.2, papers of matching for elderly care should be supplemented. There are some special issues on this topic recent years, such as "Smart Technology-Supported Independent Living for Older Adults" in International Journal of Human-Computer Interaction in 2023.

**Author response:** Thank you very much for reviewing our paper and providing valuable feedback. We sincerely appreciate your thorough evaluation, which has provided us with valuable insights to improve the quality of our work.

As suggested by the referee, we added in Section 2.2 four pieces of literature on matching documents for elderly care and some particular issues that have arisen in recent years on this topic. *[1] Zhou J, Smart technology-supported independent living for older adults: an editorial, International Journal of Human–Computer Interaction, 39 (2023), 961-963. https://doi.org/10.1080/10447318.2023.2170518.*

*[2] Liu N, Pu Q, Shi Y, et al, Older adults’ interaction with intelligent virtual assistants: the role of information modality and feedback, International Journal of Human–Computer Interaction, 39 (2023), 1162-1183. https://doi.org/10.1080/10447318.202 2.2074667.*

*[3] Wen P, Chen M, A new model for elderly emotional care routing and scheduling with multi-agency and the combination of nearby services, International Journal of Human–Computer Interaction, 39 (2023), 1111-1120. https://doi.org/10.1080/10447318.202 2.2050544.*

*[4] Sun X, Ding J, Dong Y, et al, A survey of technologies facilitating home and community-based stroke rehabilitation, International Journal of Human–Computer Interaction, 39 (2023), 1016-1042. https://doi.org/10.1080/10447318.2022.2050545.*The changes have been marked in revision mode, and the revised content is also indicated in red in the manuscript, which we have attached for your convenience.

**Reviewer #2, Concern #6:** What are the differences between heavy and light black lines in Fig. 1?

**Author response:** Thank you very much for reviewing our paper and providing valuable feedback. We sincerely appreciate your thorough evaluation, which has provided us with valuable insights to improve the quality of our work.

In response to the reviewers' feedback, we've added some descriptions of figure 1 in the "Problem description" section. In figure 1, the thin arrow indicates that both the elderly and the service provider are satisfied with each other; the thick arrow indicates that both are matched, that is, satisfy each other's requirements and satisfy the highest overall satisfaction.

The changes have been marked in revision mode, and the revised content is also indicated in red in the manuscript, which we have attached for your convenience.

**Reviewer #2, Concern #7:** “...elder people have inelastic expectations about gender indicators.” It is very important to give the reason for this claim, since it's the basis to classify "Gender" into the group of inelastic expectations.

**Author response:** Thank you for your valuable feedback and for taking the time to review our manuscript. We sincerely appreciate your thorough evaluation, which has provided us with valuable insights to improve the quality of our work.

According to referee’s comments, our reasoning for this assertion is based on online and offline survey statistics, with additional references.

*[1] Zuo M. Intelligent service and operation for the aged, Beijing: Tsinghua University Press, 2022. ISBN：9787302595328.*

The changes have been marked in revision mode, and the revised content is also indicated in red in the manuscript, which we have attached for your convenience.

**Reviewer #2, Concern #8:** A comparison with other current matching methods under the same circumstances should be given to validate the outperformance of the proposed method.

**Author response:** Thank you for your valuable feedback and for taking the time to review our manuscript. We sincerely appreciate your thorough evaluation, which has provided us with valuable insights to improve the quality of our work.

According to referee’s comments, the section "6. Comparison" has been incorporated into the body of the paper. When using the conventional two-sided matching method, neither side's needs can be satisfied nor service resources will be wasted. The suggested approach is more focused, better able to represent the unique needs of the elderly, and places more emphasis on the elderly-centered service concept than the conventional two-sided matching method.

The changes have been marked in revision mode, and the revised content is also indicated in red in the manuscript, which we have attached for your convenience.

**Reviewer #2, Concern #9:** Future research should be presented in Conclusion.

**Author response:** Thank you for your valuable feedback and for taking the time to review our manuscript. We sincerely appreciate your thorough evaluation, which has provided us with valuable insights to improve the quality of our work.

According to referee’s comments, in the conclusions section, we have included a discussion of the study's limitations and future directions. In order to examine the impact of additional behavioral factors on the outcomes of person matching, we will take into account psychological and behavioral factors in addition to the behavioral ones when matching senior citizens with service providers.

The changes have been marked in revision mode, and the revised content is also indicated in red in the manuscript, which we have attached for your convenience.
